# Celastrol improves self-renewal and differentiation of human tendon-derived stem cells by suppressing Smad7 through hypoxia

**DOI:** 10.1186/s13287-017-0724-x

**Published:** 2017-12-04

**Authors:** Tianyi Wu, Shenghe Liu, Gen Wen, Jia Xu, Yaling Yu, Yimin Chai

**Affiliations:** Department of Orthopaedic Surgery, Shanghai Jiao Tong University Affiliated Sixth People’s Hospital, 600 Yishan Road, Shanghai, 200033 People’s Republic of China

**Keywords:** Celastrol, Hypoxia, Human tendon-derived stem cells, Smad7, Multi-differentiation

## Abstract

**Background:**

We aimed to evaluate the potential enhancing effect of celastrol on the stemness of human tendon-derived stem cells (hTSCs) in vitro and the underlying molecular mechanisms.

**Methods:**

The capability of hTSC self-renewal was assessed by cell proliferation and colony formation as determined with the CCK-8 kit. Adipogenesis, chondrogenesis, and osteogenesis were determined by Oil Red O, Alcian Blue, and Alizarin Red staining, respectively. The relative mRNA levels of Sox9, PPARγ, Runx2, Smad7, and HIF1α were determined by real-time polymerase chain reaction (PCR). The levels of Smad7 and HIF1α protein were measured by immunoblotting. The chromatin immunoprecipitation (ChIP) assay was used to assess the direct binding of HIF1α to the Smad7 promoter. Suppression of Smad7 induced by hypoxia was examined using the luciferase reporter assay.

**Results:**

We found that treatment with celastrol resulted in improvement in both the multi-differentiation potential and self-renewal capability of hTSCs. Celastrol elicited hypoxia and subsequently suppressed the expression of Smad7 through direct association with the hypoxia response element consensus sequence. Further, we demonstrated that both Smad7 and HIF1α were involved in the beneficial effects of celastrol on the differentiation and self-renewal of hTSCs.

**Conclusions:**

We demonstrated the positive effect of celastrol on the stemness of hTSCs and elucidated the essential role of the HIF1α-Smad7 pathway in this process.

## Background

Clinically, it is less likely to have injuries of the bone-tendon junction (BTJ); however, the recovery phase of this complication is relatively time-consuming and its prognosis is poorer than injuries at the bone-bone or tendon-tendon junctions [1]. Despite great effort being invested for the development of therapeutics for BTJ, the overall progress in treating this anatomical abnormality remains far from adequate [2]. Poor fibrocartilage zone reconstruction and angiogenesis are often associated with insufficient healing of BTJ [3]. It has been shown that during the reconstruction phase the fibrous tissue deposited between the bone and the tendon can cause severe compromise to the mechanical capacity at the junctions recovering from BTJ [4].

In recent studies, human tendon-derived stem cells (hTSCs) have been identified in tendon tissues, with characteristics including a high proliferative index, clonogenicity, non-immunogenicity, immunosuppression, and multi-differentiation potential [5]. A growing body of evidence supports the potential therapeutic value of hTSCs in regeneration of the tendon, and thus intensive investigations have focused on the characterization, isolation, expansion, and differentiation of hTSCs in vitro [6–8]. Meanwhile, these findings from basic biological research are being proactively translated into clinical applications [9].

It is noteworthy that hypoxia, which has been reported to be involved in chondrogenesis, osteogenesis, and angiogenesis, appears to play an essential role in the differentiation and proliferation of hTSCs in vitro as well as in vivo [10]. Lee et al*.* found that hypoxia was advantageous for efficient expansion of cultured hTSCs for in vitro tendon tissue engineering [11]. In addition, hypoxia was suggested as a niche factor for the stemness of hTSCs, and was beneficial for hTSC culture and subsequent cell therapy for tendon injuries [12]. Due to the critical role of hypoxia in hTSC biology, an agent that is able to induce hypoxia in hTSCs is likely to contribute to the proliferation and differentiation of the stem cell culture and potentially to subsequent stem cell therapy for tendon repair.

Celastrol, a natural herbal product extracted from the *Tripterygium wilfordii Hook.f.* (“Thunder of God Vine”), has been shown to exhibit a variety of biological effects [13]. Of particular interest to our current study, celastrol was able to stimulate hypoxia and enhance hypoxia-inducible factor-1α (HIF1α) protein synthesis [14]. We therefore hypothesized that celastrol may potentially exert enhancing effects on hTSCs in vitro, and findings from the current study may provide insights for further investigation with animal models and eventually clinical trials and applications to treat this disorder. Here, we first obtained hTSCs isolated from tendon tissues of patients as previously described [11], and conducted comprehensive characterization in terms of the self-renewal capacity and multi-differentiation potential of these hTSCs. To the best of our knowledge, our findings here provide the first evidence that celastrol significantly enhances the stemness of hTSCs in vitro. Additional analysis revealed that the beneficial effects of celastrol involve the induction of hypoxia. Furthermore, using a bioinformatics approach, we hypothesized and experimentally confirmed Smad7, which was negatively correlated to the proliferation and differentiation of TSCs [15], as a novel target of HIF1α in this process. Stable silencing of HIF1α mediated via shRNA and lentiviral-mediated stable overexpression of Smad7 in hTSCs fully abolished the stimulatory effect of celastrol treatment on both the multi-differentiation potential and self-renewal ability, thereby suggesting the involvement of the HIF1α- Smad7 pathway in such an effect.

## Methods

### Isolating and culturing of hTSCs

The experimental protocols were approved by the Institutional Ethics Committee. Human patellar tendons were obtained from patients who received anterior cruciate ligament reconstruction with bone-patellar tendon-bone autograft and who provided written informed consent. The peritendinous connective tissue was carefully dissected, and small pieces of tendon tissues were digested by type I collagenase (4 mg/ml; Sigma, St. Louis, MO, USA) in sterile phosphate-buffered saline (PBS) at 37 °C for 2 h. The suspension of single cells was prepared by passing through a cell strainer (70 μm) followed by two rinses with PBS. The dissociated hTSCs were cultured in complete low-glucose Dulbecco’s modified Eagle’s medium (DMEM) supplemented with 1% penicillin-streptavidin-glutamine (PSG) and 10% fetal bovine serum (FBS; Gibco, Grand Island, NY, USA) at a density of 500/cm^2^ in a humidified incubator supplied with 5% CO_2_ to allow colony formation. At day 3, PBS was used to wash off the non-adherent cells and the remaining cells were trypsinized at day 7 for passage.

### Colony formation assay

A total of 100 hTSCs were seeded into a 10-cm petri dish and continually cultured for up to 3 weeks. Colonies that formed were visualized under a light microscope after staining with 0.5% crystal violet (Sigma, St. Louis, MO, USA) for 15 min, and colonies with a diameter above 2 mm were counted.

### Proliferation assay

The proliferation of hTSCs was determined using the CCK-8 kit (Dojindo, Kumamoto, Japan). In brief, hTSCs were placed in a 96-well plate to receive various treatments; 10 μl CCK-8 solution was added to each well followed by the chromogenic reaction for 15 min at 37 °C. Recordings of the absorption at 450 nm were performed with a microplate reader (Molecular Devices, Sunnyvale, CA, USA) to calculate the relative cell viability.

### Multi-differentiation assays

The differentiation capacity of hTSCs in terms of osteogenesis, chondrogenesis, and adipogenesis following celastrol treatment was assessed by multi-differentiation assay. The hTSCs were maintained under the following conditioned media: osteogenic induction medium (DMEM-low glucose, 50 mg/ml ascorbic 2-phosphate, 20% FBS, 50 mg/ml insulin-transferrin-selenious acid mix, and 100 mg/ml sodium pyruvate), chondrogenic induction medium (DMEM, 0.2 mM ascorbic 2-phosphate, 20% FBS, and 10 mM glycerol 2-phosphate), and adipogenic induction medium (DMEM, 100 mM indomethacin, 20% FBS, and 0.5 mM isobutylmethylxanthine). Adipogenic differentiation was assessed based on Oil Red O (Millipore, Billerica, MA, USA) staining. Calcium deposition was measured with the use of Alizarin Red solution (Millipore, Billerica, MA, USA). Chondrogenesis was evaluated using Alcian Blue (Millipore, Billerica, MA, USA) staining.

### Quantitative real-time polymerase chain reaction (qRT-PCR)

Trizol reagent (Invitrogen, Carlsbad, CA, USA) was used to extract the total RNA according to the provided protocol. The quantity and quality of the RNA product was first examined by BioAnalyzer 2100 (Agilent, Santa Clara, CA, USA). Synthesis of the cDNA was performed by the PrimerScript 1st Strand cDNA Synthesis Kit (Clontech, Mountain View, CA, USA) in accordance with the manufacturer’s instructions. The relative mRNA levels of Sox9, PPARγ, Runx2, Smad7, and HIF1α were determined by the TaqMan Assay using GAPDH as the internal control. Primers used in this study were: SOX9: forward 5′-GTA CCC GCA CTT GCA CAA-3′, reverse 5′-TCT CGC TCT CGT TCA GAA GTC-3′; PPARγ: forward 5′-CGT GGC CGC AGA TTT GAA-3′, reverse 5′-CTT CCA TTA CGG AGA GAT CCA-3′; Runx2: forward 5′-TCT TAG AAC AAA TTC TGC CCT-3′, reverse 5′-TGC TTT GGT CTT GAA ATC ACA-3′; Smad7: forward 5′-GAA TCT TAC GGG AAG ATC AAC-3′, reverse 5′-CGC AGA GTC GGC TAA GGT-3′; HIF1α: forward 5′-AGG TGG ATA TGT CTG GGT-3′, reverse 5′-AAG GAC ACA TTC TGT TTG TTG-3′; GAPDH: forward 5′-CTG ACT TCA ACA GCG ACA CC-3′, reverse 5′-TAG CCA AAT TCG TTG TCA TAC-3′.

### Western blot

The cells were lysed in radio-immunoprecipitation assay lysis buffer and the concentration of protein was determined using the bicinchoninic acid Protein System Assay. Protein samples of equal quantity were separated by SDS-PAGE and transferred to polyvinylidene difluoride membrane on ice. The membrane was subsequently blocked with 5% skimmed milk in TBST buffer (Tris-buffered saline with 0.05% Tween-20) for 1 h at room temperature, and followed by incubation with the primary antibodies (Smad7, Abcam #90086, 1:500; HIF1α, CST #3716, 1:1000; GAPDH, CST #2118, 1:2000) overnight at 4 °C. After a thorough rinse with TBST, the membrane was hybridized to horseradish peroxidase-conjugated secondary antibody (anti-rabbit, CST #7074, 1:5000) for 1 h at room temperature, and then rinsed with TBST again for 30 min. The enhanced chemiluminescence (ECL; Millipore, Billerica, MA, USA) method was employed to visualize the final blots, with GAPDH as the loading control.

### Luciferase assay

Wild-type or hypoxia response element (HRE)-mutated promoter region of Smad7 was cloned into pGL4 luciferase reporter plasmid and subsequently transfected into hTSCs using Lipofectamine 2000 (Invitrogen, Carlsbad, CA, USA). Cells were treated with or without cobalt chloride to establish hypoxic or normoxic conditions, respectively. The Bright-Glo Luciferase Assay System (Promega, Madison, WI, USA) was used to determine the relative luciferase activity.

### Chromatin immunoprecipitation (ChIP)

hTSCs that were growing exponentially under either hypoxic or normoxic conditions were fixed with 4% formaldehyde for 1 h, followed by ultrasonication on ice to obtain DNA fragments with a length of around 800–1000 bp. The DNA fragments were immunoprecipitated with HIF1α antibody (CST #3716, Cell Signaling Technologies, Danvers, MA, USA) and recovered using high-salt wash buffer. Binding efficiency (Bound/RPII%) was defined as the ratio of amplification efficiency between HIF1α and polymerase RNA II. The sequences of primers used in the ChIP assay are available upon request.

### Stable cell lines

HIF1α stable knockdown cell lines were established by integration of specific shRNA obtained from Sigma library targeting HIF1α (SHCLNV-NM_001530), which was packaged to transduce hTSCs following the manufacturer’s instructions. Stable overexpression of Smad7 was introduced in hTSCs using the Lenti-X Expression System (Clontech, Mountain View, CA, USA). Briefly, the Smad7 coding sequence without the endogenous promoter region was cloned into Lenti-X vector using standard molecular cloning protocols. The Lenti-X Smad7 vector, as well as an empty Lenti-X vector as a negative control, was packaged to transduce hTSCs following the manufacturer’s instructions.

### Statistical analysis

Variance analysis of results from at least three independent experiments were preformed using SPSS 23 software, and all the data are presented as mean ± standard deviation (SD). Student’s *t* test was utilized for pairwise comparison; *p* values less than 0.05 were considered statistically different.

## Results

### Celastrol improves the self-renewal ability of hTSCs

**Fig. 1:**
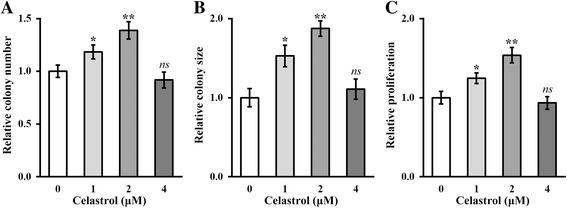
Celastrol improves the self-renewal ability of hTSCs. Colony number (**a**), colony size (**b**), and proliferation (**c**) of hTSCs after treatment with increasing celastrol concentrations (0, 1, 2, and 4 μM). Data are shown as mean ± SD. Student’s *t* test was utilized for pairwise comparison. *p < 0.05, **p < 0.01, versus 0 μM celastrol. *ns* not significant,

Accumulating evidence supports the crucial role of hypoxia in the proliferation and differentiation of TSCs. Interestingly, the use of celastrol to induce hypoxia has been reported recently, which prompted us to examine the potential influence of celastrol on the hTSCs through induction of hypoxia. Here, we first treated hTSCs with different doses of celastrol (0, 1, 2, and 4 μM) for 24 h, and then evaluated their self-renewal capacity based on both proliferation and colony formation assays. We found that the relative number, as well as the average size, of the colonies that formed were substantially increased as a result of celastrol treatment up to 2 μM (Fig. 1a and b; *p* < 0.05). In a similar way, the cell viability clearly revealed that celastrol markedly promoted the proliferation of hTSCs (Fig. 1c; *p* < 0.05). However, a higher dose of celastrol (4 μM in our system) resulted in slight suppression of both cell proliferation and colony formation (Fig. 1a–c). Therefore, 2 μM was determined as the optimal dose of celastrol for the subsequent experiments in the current investigation. To the best of our knowledge, our data here are the first to report that celastrol improves the stemness of patient-derived hTSCs.

### Celastrol improves the differentiation potential of hTSCs

**Fig. 2:**
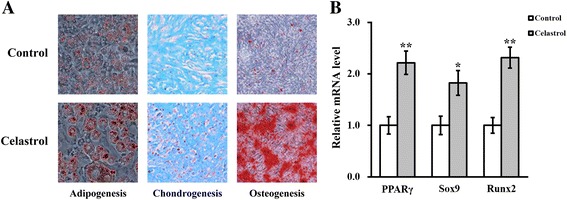
Celastrol improves the differentiation potential of hTSCs. **a** At day 14 after differentiation induction in the absence (control) or presence of 2 μM celastrol, the extent of adipogenesis, chondrogenesis, and osteogenesis was evaluated by Oil Red O, Alcian Blue, and Alizarin Red S staining assays, respectively. **b** At day 14 after differentiation induction in the absence (control) or presence of 2 μM celastrol, the extent of adipogenesis, chondrogenesis, and osteogenesis were evaluated by mRNA levels of the adipogenic marker PPARγ, the chondrogenic marker Sox9, and the osteogenic marker Runx2. Data aere shown as mean ± SD. Student’s *t* test was utilized for pairwise comparison. *p < 0.05, **p < 0.01, versus control

Our results demonstrated that celastrol treatment improved the self-renewal capacity of hTSCs in vitro. We next sought to examine the potential influence of celastrol on the multi-differentiation potential of hTSCs. To achieve this aim, three distinct differentiation directions, namely chondrogenesis, osteogenesis, and adipogenesis, were interrogated upon treatment with celastrol. As demonstrated in Fig. 2a, Oil Red O staining revealed a dramatic increase in adipogenesis in hTSCs treated with celastrol. Similar changes were observed regarding osteogenesis and chondrogenesis as evidenced by staining with Alizarin Red solution and Alcian Blue, respectively. Further validation of these phenotypic alterations at the molecular level was obtained by assessments of specific biomarkers. Treatment with celastrol greatly upregulated PPARγ (a marker for adipogenesis), Runx2 (a marker for osteogenesis), and Sox9 (a marker for chondrogenesis) (Fig. 2b; *p* < 0.05). These findings clearly show that celastrol promoted the multi-differentiation potential of hTSCs in addition to its effect on self-renewal capacity.

### Celastrol induces hypoxia and downregulates Smad7

**Fig. 3:**
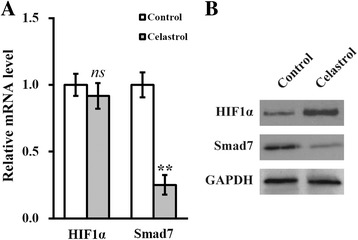
Celastrol induces hypoxia and downregulates Smad7 expression. **a** mRNA and **b** protein levels of HIF1α and Smad7 were analyzed in hTSCs cultured in the absence (control) or presence of 2 μM celastrol. Data are shown as mean ± SD. Student’s *t* test was utilized for pairwise comparison. **p < 0.01, versus control. *ns* not significant

Our previous observations were in line with the hypothesis that treatment with celastrol could benefit the stemness of hTSCs in vitro. Next, we aimed to address whether celastrol-induced hypoxia was involved in this process. First, we examined the expression of HIF1α and Smad7, a target of HIF1α, at both the mRNA and protein levels following celastrol treatment. As shown in Fig. 3a, HIF1α expression at the transcriptional level was unaltered in hTSCs treated with celastrol. In contrast, there was a remarkable downregulation of Smad7 mRNA induced by celastrol (*p* < 0.05). Moreover, Western blot results revealed a significant upregulation in the protein level of HIF1α and downregulation of Smad7 (Fig. 3b). Altogether, these data suggested that celastrol exerted a post-transcriptional regulation of HIF1α expression, whereas Smad7 was modulated at the mRNA level. Our results agree with the canonical regulatory mode in which celastrol induced an hypoxic environment which in turn inhibited the degradation of HIF1α mediated via the ubiquitin-proteasome system and thereby stabilized the HIF1α protein.

### Expression of Smad7 is downregulated by hypoxia in hTSCs

**Fig. 4:**
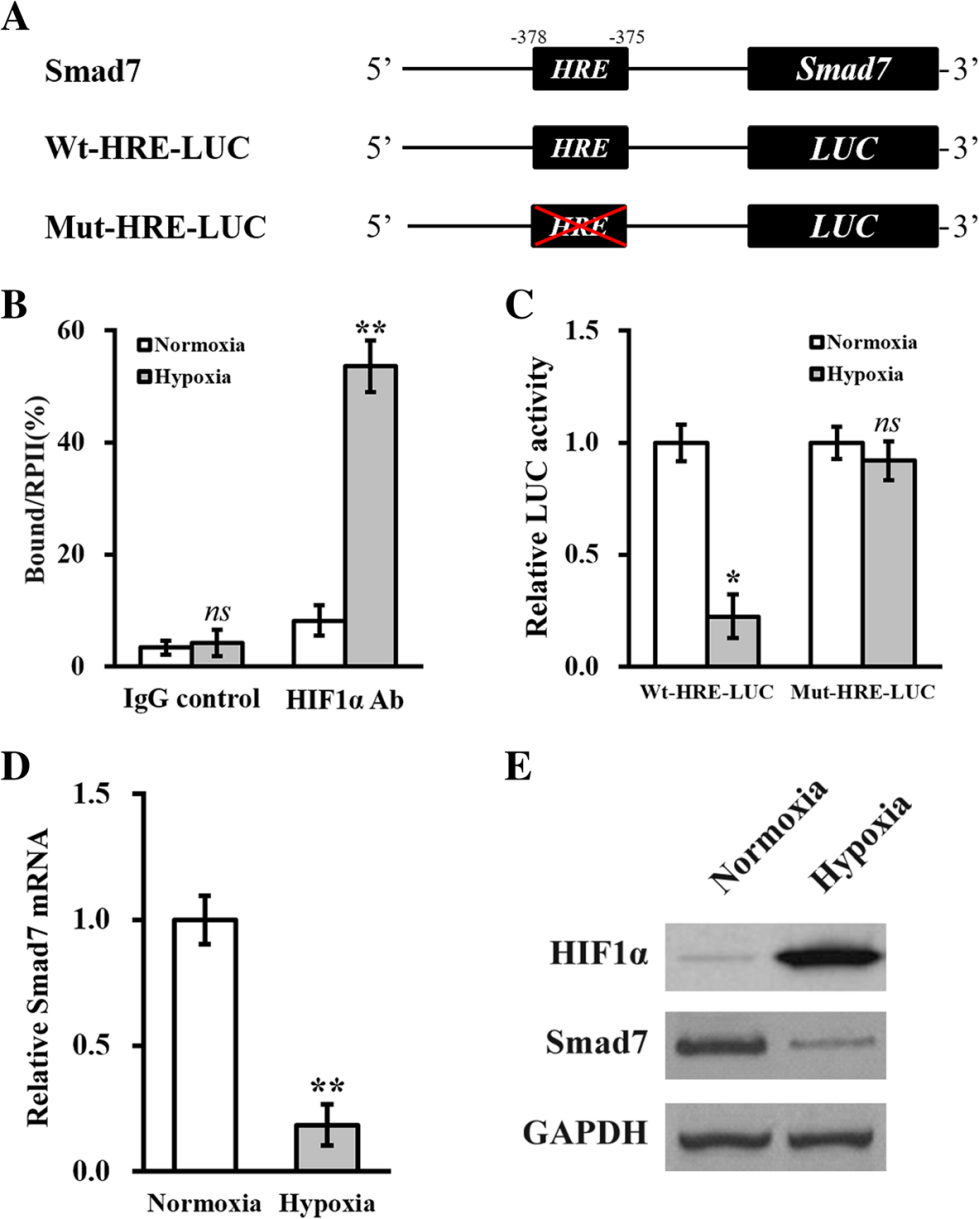
Expression of Smad7 is downregulated by hypoxia in hTSCs. **a** The predicted hypoxia response elements (*HRE*) were identified in the promoter region of Smad7. Wild type (*Wt-HRE-LUC*) and mutated (*Mut-HRE-LUC*) HRE sequences from Smad7 promoter were cloned, respectively, to the 5′-prime of the luciferase reporter (*LUC*). **b** hTSCs incubated with media containing 0 (normoxia) or 100 μM (hypoxia) CoCl_2_, respectively, were analyzed by ChIP assay using IgG control or HIF1α antibody (*Ab*). **c** LUC activities of Wt-HRE-LUC or Mut-HRE-LUC constructs described in **a** were measured in hTSCs under normoxic or hypoxic conditions, respectively. mRNA (**d**) and protein (**e**) levels of Smad7 were analyzed in hTSCs under normoxic or hypoxic conditions, respectively. Data are shown as mean ± SD. Student’s *t* test was utilized for pairwise comparison. *p < 0.05, **p < 0.01, versus normoxia. *ns* not significant

We so far have unambiguously shown that treatment with celastrol stabilized and upregulated HIF1α as well as the potential target gene Smad7. We went on to experimentally confirm that Smad7 was suppressed by celastrol-induced hypoxia in hTSCs. Through close inspection of the promoter region of Smad7, we identified the candidate HRE consensus sequence [16], as illustrated in Fig. 4a. The direct binding of HIF1α to the HRE region on Smad7 promoter was confirmed by ChIP assay, which showed significantly enriched HRE in HIF1α-immunoprecipitate compared with IgG control (*p* < 0.01). This association was potentiated by the hypoxic environment generated by cobalt chloride application (Fig. 4b). Furthermore, to establish a quantitative model for the regulatory action of hypoxia on Smad7, we constructed luciferase reporter plasmids carrying either wild-type or HRE-mutated Smad7 promoter. Hypoxia dramatically repressed luciferase activity driven by the wild-type Smad7 promoter. However, mutation of the HRE sequence in Smad7 promoter readily abolished such an effect (Fig. 4c; *p* < 0.05), suggesting the essential role of HRE in the downregulation of Smad7 induced by hypoxia. Most importantly, we validated this regulatory mechanism regarding Smad7 in hTSCs. CoCl_2_-simulated hypoxia treatment greatly reduced the Smad7 transcript level (Fig. 4d; *p* < 0.01). Consistently, our immunoblotting results revealed similar changes for Smad7 protein (Fig. 4e). The efficiency of CoCl_2_-induced hypoxia was evidenced by the stabilization of the HIF1α protein (Fig. 4e). Taken together, our results demonstrate that the production of Smad7 was suppressed by hypoxia in hTSCs.

### HIF1α and Smad7 are implicated in the enhancing effects of celastrol on hTSC self-renewal and differentiation

**Fig. 5:**
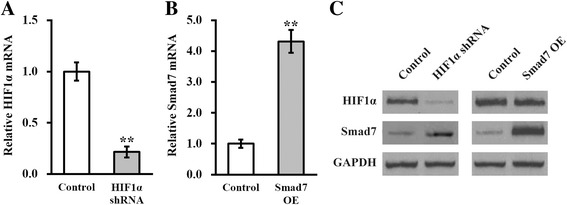
Stable HIF1α knockdown and Smad7 overexpression in hTSCs. mRNA levels of HIF1α (**a**) and Smad7 (**b**) were examined in hTSCs following their stable knockdown and overexpression, respectively. **c** Protein levels of both HIF1α and Smad7 were examined in hTSCs following stable knockdown of either HIF1α or Smad7, respectively. Data are shown as mean ± SD. Student’s *t* test was utilized for pairwise comparison. **p < 0.01, versus respective control

**Fig. 6:**
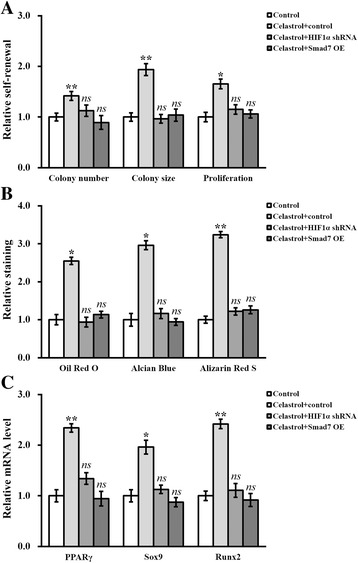
HIF1α and Smad7 are implicated in the enhancing effects of celastrol on hTSC self-renewal and differentiation. **a** Colony number, colony size, and proliferation of hTSCs in the absence (control) or presence of 2 μM celastrol, plus HIF1α knockdown and Smad7 overexpression, respectively. **b** At day 14 after differentiation induction in the absence (control) or presence of 2 μM celastrol, plus HIF1α knockdown and Smad7 overexpression, the extent of adipogenesis, chondrogenesis, and osteogenesis was evaluated by Oil Red O, Alcian Blue, and Alizarin Red S staining assays, respectively. **c** At day 14 after differentiation induction in the absence (control) or presence of 2 μM celastrol, plus HIF1α knockdown and Smad7 overexpression, the extent of adipogenesis, chondrogenesis, and osteogenesis were evaluated by mRNA levels of the adipogenic marker PPARγ, the chondrogenic marker Sox9 and the osteogenic marker Runx2. Data are shown as mean ± SD. Student’s *t* test was utilized for pairwise comparison. *p < 0.05, **p < 0.01, versus control. *ns* not significant

Our previous observations showing the celastrol-elicited hypoxia and subsequent downregulation of Smad7 in hTSCs prompted us to address whether the enhancing effect of celastrol on the stemness of hTSCs was mechanistically mediated by HIF1α and Smad7. To this purpose, we first established stable HIF1α knockdown and Smad7 overexpression hTSC lines, respectively. The efficiency of HIF1α knockdown and Smad7 overexpression were evaluated by the use of qRT-PCR (Fig. 5a and b; *p* < 0.01) and immunoblotting, respectively (Fig. 5c). Consistent with our previous conclusion, Smad7 protein was significantly upregulated in the hTSCs lacking HIF1α. Either deficiency of HIF1α or overexpression of Smad7 completely abolished the stimulatory effect of celastrol on promoting the colony formation of hTSCs, as indicated by the number and average size of colonies (Fig. 6a; *p* < 0.05). Similarly, celastrol-induced proliferation of hTSCs was markedly blunted by either HIF1α knockdown or Smad7 overexpression (Fig. 6a). The enhancement of the multi-differentiation potential in hTSCs resulting from celastrol treatment was inhibited by both HIF1α knockdown and Smad7 overexpression (Fig. 6b), accompanied by a decrease in the expression of the respective molecular markers (Fig. 6c). These results demonstrated that celastrol-elicited improvement in hTSC self-renewal and differentiation required the participation of HIF1α and Smad7.

## Discussion

A growing body of evidence supports the possibility that celastrol could generate an hypoxic environment [14], and this has been widely studied in the self-renewal/proliferation and multi-differentiation of hTSCs [17]. In the current investigation, we hypothesized that celastrol may exert beneficial effect on hTSCs through induction of hypoxia. To verify this hypothesis, we isolated hTSCs from individuals who received anterior cruciate ligament reconstruction with bone-patellar tendon-bone autograft. Our findings suggested that treatment with celastrol stimulated the proliferation, self-renewal, and multi-differentiation of hTSCs in a dose-dependent manner, with a slight adverse effect at the highest concentration. The multi-differentiation potential of hTSCs following celastrol treatment was assessed in terms of adipogenesis, chondrogenesis and osteogenesis by both morphological examination and detection of molecular biomarkers, in all of which celastrol exerted significant stimulatory effects. In our experiments, the celastrol-induced hypoxia was consolidated via the accumulation of HIF1α. A specific knockdown assay further validated the pivotal function of HIF1α in mediating the pro-stemness effect of celastrol on hTSCs. Most importantly, here we identified a downstream effector, Smad7, that was regulated by HIF1α at the transcriptional level and played a critical role in this process. Our findings for the first time present evidence for the potential of celastrol to improve the stemness of hTSCs in vitro, a process involving the HIF1α-Smad7 axis, which immediately necessitates further investigations using animal disease models and in clinical trials. Celastrol, as the active component from a variety of herbs, has been extensively studied in various therapeutic applications [13]. The molecular studies looking into the pharmacological properties of celastrol identified diverse potential targets, most of which involved the IKK-NF-κB pathway. For instance, celastrol has been shown to directly suppress the IKKα and β kinase through targeting Cys-179 in the activation loop of IKKβ [18]. High-throughput screening for inhibitors of androgen signaling has also identified celastrol as blocking the interactions between co-chaperone Cdc37/p23 and HSP90 [19]. Yang et al*.* reported the inhibition of rabbit 20S proteasomes by celastrol which elicited cell apoptosis of prostate cancer cells [20]. Additionally, treatment with celastrol triggered a heat shock response by induction of HSP70 expression [21]. However, the potential effect of celastrol on tendon differentiation has not been fully understood. Here, we used hTSCs prepared from human patellar tendon and confirmed that celastrol was sufficient to improve self-renewal and multi-differentiation potential simultaneously.

Recent investigations revealed the hypoxia-eliciting property of celastrol [14]. Han et al. found that, in multiple cancer cell lines, celastrol could induce accumulation of HIF1α protein, which subsequently entered the nucleus and promoted the transcription of the HIF1 target genes [14]. Consistent with results in our current study, celastrol did not affect transcription of HIF1α, but instead induced the accumulation of the HIF1α protein. In addition, our findings clearly showed that treatment with celastrol also elicited hypoxia in the dissociated hTSCs, and supported the post-transcriptional regulation in agreement with the canonical stabilizing function of HIF1α. It is also worth pointing out that despite the apparent critical role of hypoxia in mediating the stimulatory effects of celastrol on hTSCs, the downstream molecular mechanisms remain elusive.

The Smad family of proteins, a subgroup of intracellular proteins, transduce extracellular signals such as transforming growth factor (TGF)-β to the nucleus to induce the activation of downstream gene transcription [22]. There are three categories of Smad proteins: receptor-regulated Smads (r-Smad), common-mediator Smads (co-Smad) that interact with r-Smad in various signaling pathways, and inhibitory Smads (i-Smad) [23–25]. Smad6 and Smad7 are two i-Smads that function to block the activation of r-Smads and co-Smads [25]. In human umbilical vein endothelial cells, celastrol could suppress the TGF-β1/Smad signaling pathway [26], and the Smad7 protein is likely involved in this action of celastrol [27]. In the context of tendon differentiation, Smad7 was found to be not only the key regulator of the TGF-β/BMP signaling pathway, but also a key factor in the differentiation and proliferation of TSCs, whose expression level was negatively correlated to TSC proliferation and differentiation [15]. These previous results are again verified by our present data, where Smad7 was markedly suppressed in response to the hypoxia induced by celastrol, which in turn led to enhanced proliferation and differentiation of hTSCs. In view of the wide involvement of the Smad family proteins, especially Smad7, in various cellular activities, further studies are necessary to demonstrate the identity of factors downstream of Smad7.

## Conclusions

In conclusion, we demonstrated in the current work that treatment with celastrol effectively improved the multi-differentiation potential and self-renewal capacity of hTSCs in vitro, predominately mediated via the HIF1α-Smad7 axis. Our findings suggest the potential of celastrol for clinical use in the management of tendon injury and necessitate further laboratory as well as clinical investigations.

## Abbreviations

BTJ: Bone-tendon junction
ChIP: Chromatin immunoprecipitation
DMEM: Dulbecco’s modified Eagle’s medium
FBS: Fetal bovine serum
HIF1α: Hypoxia-inducible factor-1α
HRE: Hypoxia response element
hTSC: Human tendon-derived stem cell
PBS: Phosphate-buffered saline
qRT-PCR: Quantitative real-time polymerase chain reaction
TGF: Transforming growth factor
